# Analysis of Mannose‐Binding Lectin Protein and mRNA Levels on Selected Chicken Breeds in South Africa

**DOI:** 10.1002/vms3.70045

**Published:** 2024-10-18

**Authors:** Peter Ayodeji Idowu, Takalani J. Mpofu, Oliver T. Zishiri, Khathutshelo A. Nephawe, Bohani Mtileni

**Affiliations:** ^1^ Department of Animal Sciences, Faculty of Science Tshwane University of Technology Pretoria South Africa; ^2^ Discipline of Genetics, School of Life Sciences University of KwaZulu‐Natal Durban South Africa

**Keywords:** chicken innate system, immune response, mannose‐binding lectin (MBL) detection, mannose‐binding lectin (MBL) expression, pathogens, poultry production

## Abstract

**Background:**

Mannose‐binding lectin (MBL) is a key component of the innate immune system that plays a crucial role in binding to the microbial sugar surface to recognize and eliminate pathogens by activating the complement system.

**Objective:**

To detect and quantify the MBL protein concentration and chicken MBL expression in selected chicken breeds in South Africa.

**Methods:**

Forty‐five blood samples from three indigenous chicken breeds, Ovambo (OV = 9), Venda (VD = 9) and Potchefstroom Koekoek (PK = 9), and two exotic chicken breeds, Rhode Island Red (RIR = 9) and Lohmann Brown (LB = 9), were used for MBL protein concentration using enzyme‐linked immunosorbent assay (ELISA) techniques. Also 20 liver samples from symptomatic two indigenous chicken breeds, OV (5) and PK (5), and two exotic chicken breeds, RIR (5) and LB (5), were used for MBL expression using quantitative polymerase chain reaction (qPCR) techniques. A general linear model was done using Tukey's multiple comparison post hoc test.

**Results:**

The findings revealed MBL protein concentration from 5.26 to 18.56 µg/mL. The LB breed had the lowest mean 6.40 ± 0.80 µg/mL, whereas the PK breed had the highest mean MBL concentration of 17.70 ± 0.24 µg/mL of MBL protein concentration. At 12, 25 and 35 weeks, the MBL proteins of OV, VD, PK, RIR and LB varied significantly at *p* ≤ 0.05. The mRNA MBL expression of OV and LB breeds showed a 1‐fold decrease in MBL expression, while RIR showed a 2‐fold increase in MBL expression, and the PK showed more than a 3‐fold increase in MBL expression relative to the control. The least‐squares means for OV, LB, PK and RIR mRNA MBL expression were 0.54 ± 0.19, 0.68 ± 0.30, 4.46 ± 2.76 and 2.89 ± 0.19 µg/mL, respectively.

**Conclusion:**

MBL protein was detected and quantified with distinct differences in concentration and expression levels  at the presence of *mycoplasma gallisepticum* among the sampled South African chicken breeds. This highlights the genetic diversity of MBL as a tool for disease prevention in South African chicken breeds.

## Graphical abstract

The graphical representation of mannose‐binding lectin analysis of selected South Africa chicken breeds.

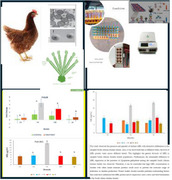

**Abbreviations**
MBL: mannose‐binding lectin
qPCR: quantitative polymerase chain reaction
TLR: Toll‐like receptors

## 1 Introduction

Globally, chicken production is one of the fastest growing agricultural industries. Precisely, from 2019 to 2020, the population of chickens increased from 178 to 180 million per head in South Africa (Farming South Africa 2023). This increase has led to an increase in the country's Gross Domestic Product, as it is a crucial source of protein, means of livelihood and a source of employment (Conan et al. 2012; Idowu, Mpayipheli, and Muchenje 2018). In general, South African indigenous chicken breeds, such as Ovambo (OV), Venda (VD) and Potchefstroom Koekoek (PK), are known for their unique characteristics, including adaptability to local environmental conditions and disease tolerance (Idowu, Zishiri, et al. 2021; Manyelo et al. 2020; Mpenda et al. 2019; Mtileni et al. 2021). On the other hand, exotic breeds like the Rhode Island Red (RIR) and Lohmann Brown (LB) are hard dual‐purpose chickens, primarily used for both egg production and meat (Lohmann Breeder 2019; Livestock Conservancy 2020).

In recent years, there has been a growing interest in exploring the genetic and immune system components of South African chicken breeds (Khanyile, Dzomba, and Muchadeyi 2015; Mpenda et al. 2019; Mtileni et al. 2012). Mpenda et al. (2019) and Azimu et al. (2018) reported cases of individual heterogeneity in resistance and susceptibility to diseases among chicken breeds. Moreover, Smith, Powers, and Beal () suggested that understanding the interactions between chicken breeds and their immune systems is crucial for selecting chickens for disease resistance, which will promote overall health and productivity (Berghof et al. 2019; Smith, Powers, and Beal).

One unique aspect of the immune system in chickens is the presence of lectins, which are proteins that play a crucial role in innate immunity (Drickamer and Taylor 2015). The mannose‐binding lectin (MBL) is an important pattern recognition receptor (PRR) involved in the recognition and elimination of pathogens by binding to specific sugar molecules (such as mannose, *N*‐acetylmannosamine, *N*‐acetylglucosamine, glucose and fucose) on pathogen surfaces (Howard, Farrar, and Sacks 2018; Smith and Fiddaman 2022). Additionally, MBL acts as an opsonin, enhancing the phagocytosis of pathogens by immune cells by activating the complement system (Gupta and Gupta 2021). Lastly, the MBL is a member of the collagenous c‐type lectin family, also referred to as ‘collectin’, a subfamily of the c‐type lectin superfamily that consists of lectins that are Ca^2+^‐dependent (Howard, Farrar, and Sacks 2018; Zhang, Van Eijk, et al. 2017). It is important to note that the primary site for MBL production is the liver, but then upregulated and released into the bloodstream at the entry of pathogens (Norup et al. 2019; Ulrich‐Lynge et al. 2015a). Ulrich‐Lynge et al. (2015a) stated that the entry of bacteria enhances the ability of the liver to produce more MBL and other inflammatory proteins.

To detect and quantify MBL protein concentration, similar studies in humans and chickens have used several methods such as reverse phase high‐pressure liquid chromatography (reverse phase HPLC) and affinity chromatography (Daniel 1987; Laursen, Nielsen, and Koch 1998). However, these methods have been discouraged due to their limitations. Kalia, Singh, and Kaur (2021) explained that reverse phase HPLC requires molecules with high affinity to influence the speed of detection, and MBL in some cases has a low binding affinity. The affinity chromatography method is faced with challenges in choosing the appropriate ligand specificity and matrix, and in some cases, this method cannot purify MBL from other serum constituents. More techniques for MBL detection were reviewed by Idowu, Idowu, et al. (2021). Recently, studies have used enzyme‐linked immunosorbent assays (ELISA) and quantitative polymerase chain reaction (qPCR) for MBL protein concentration and MBL mRNA expression, respectively (Norup et al. 2019; Ulrich‐Lynge et al. 2015a; Zhang, Bouwman, et al. 2017).

Regarding MBL binding affinity, MBL has been linked to providing immunity in chickens against bacterial and viral infections (Kjærup et al. 2013; Ulrich‐Lynge et al. 2015a). A study by Ulrich‐Lynge et al. (2015a) revealed the strong affinity of MBL for *Staphylococcus aureus*. However, one of the negative impacts of low MBL protein concentrations in chickens is increased in faecal shedding of *Salmonella enterica* serovar Montevideo (Ulrich‐Lynge et al. 2016). This suggests that low concentrations of MBL protein are positively correlated with higher salmonella prevalence in chickens. Consequently, adequate levels of MBL are crucial for maintaining optimal chicken health.

The importance of MBL has been widely studied in other species (Coumou et al. 2019; Juul‐Madsen et al. 2011; Kalia, Singh, and Kaur 2021; Mu et al. 2017; Taylor and Drickamer 2019). These studies explained the ability of MBL to phagocyte infectious disease. However, to the best of our knowledge, no research has been conducted to detect the presence and quantify MBL protein concentration and its mRNA expression in chicken breeds present in South Africa. Therefore, this study is aimed to investigate the presence of and quantify the MBL protein concentration and mRNA MBL expression in chicken breeds in South Africa. This research would contribute valuable insight into the mechanisms of resistance against poultry‐related diseases, with great knowledge on the genetic basis of innate immunity in chicken breeds available in South Africa. This research would pave the way for targeted breeding programs to enhance the overall health and productivity of chicken breeds in South Africa.

## 2 Materials and Methods

### 2.1 Study Animal Site and Animal Management

**FIGURE 1:**
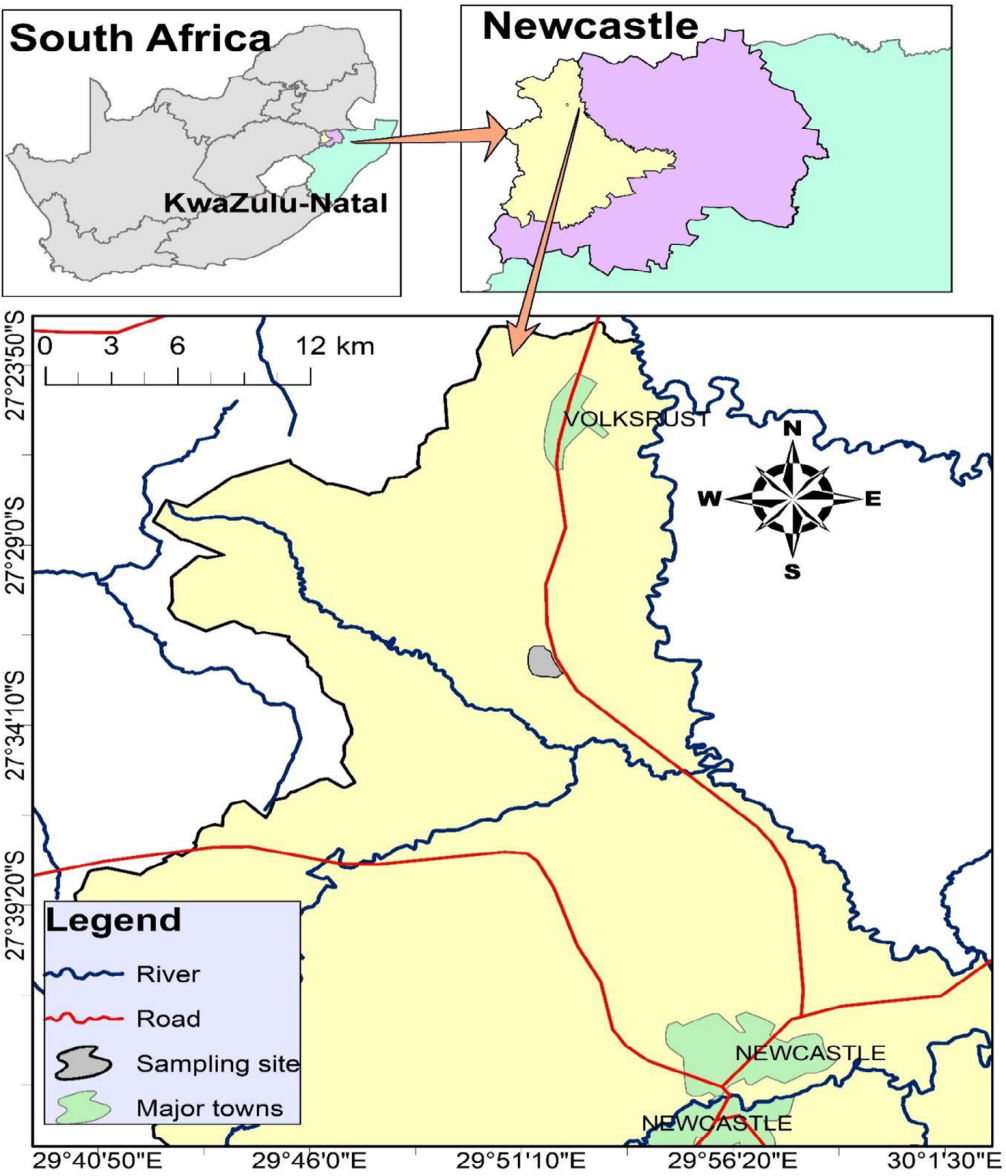
Map of the study site. The mean cMBL protein concentration of selected South African chicken breeds.

The study was conducted at a farm in Newcastle, Kwazulu‐Natal Province, South Africa, with coordinates 2732′57.3″ S 2951′58.8″ E. The farm is registered under the South African Poultry Association with strict adherence to all safety and health measures. All the chicken breeds are kept or housed in a separate pen according to different breeds and age class with a concrete floor and sawdust bedding. Standard chicken feed and water were provided ad libitum. All the animals were vaccinated according to the South Africa Poultry Association health guidelines (Figure 1).

### 2.2 Animal Material and Tissue Collection

Forty‐five blood samples were randomly collected from three indigenous chicken breeds, OV (9), VD (9) and PK (9), and two commercial chicken breeds available in the chicken farm, RIR (9) and LB (9), for MBL protein concentration. Furthermore, 20 liver samples were randomly collected from 5 symptomatic birds of each breed showing symptoms of *Mycoplasma gallisepticum*, whereas liver samples from chickens with no symptoms of Mycoplasma were used as controls, 3 per breed, 2 indigenous chicken breeds, OV and PK, and 2 commercial chicken breeds, RIR and LB, per age group, to investigate variation in MBL mRNA expression. Blood was drawn and allowed to coagulate at room temperature for approximately 2 h before centrifugation and removal of serum. The liver samples were collected and placed into a sterile container and placed into a dry ice in a cooler for easy transportation and to maintain the integrity of the samples.

### 2.3 DNA Extraction and PCR Amplification

The liver samples were homogenized by bead bashing protocols according to the DNA/RNA Shield specifications. Then, 0.5 volumes were added to the solid tissue buffer, and 10 µL of proteinase K was added to the lysate. The mixture in the tube was vortexed for 10–15 s and then incubated at 55°C for 3 h. To remove insoluble debris, the sample was centrifuged at ≥12,000 × *g* for 1 min. The aqueous supernatant was subsequently transferred to a clean microcentrifuge tube. One volume of genomic binding buffer was subsequently added to the digested sample, which was subsequently vortexed for 10–15 s. The mixture was then transferred to a Zymo‐Spin IIC‐XLR column in a collecting tube and centrifuged at ≥12,000 × *g* for 60 s. The flow through was subsequently discarded with the collecting tube. After which, 400 µL of g‐DNA pre‐wash, 700 µL of g‐DNA wash buffer and 200 µL of g‐DNA wash buffer were added to the spin column in a new collecting tube, which was subsequently centrifuged at ≥12,000 × *g* for 1 min. The flow through and the collecting tube were then emptied. This suspension was subjected to DNA extraction using the Quick‐DNA Miniprep Plus Kit (Zymo Research, Irvine, CA, USA) following the manufacturer's instructions for tissue samples. The quality and concentration of the RNA samples were measured using a NanoDrop One Microvolume UV–Vis Spectrophotometer (A260/A280 ratio around ∼2.0 [Thermo Fisher Scientific]). The cDNA strand was synthesized using a LunaScript RT SuperMix Kit (New England Biolabs, Ipswich, MA, USA) using 1 µg of 2–4 µL of RNA template, 4 µL of LunaScript RT SuperMix (5×) with and 20 µL of nuclease‐free water were used according to the manufacturer instructions. The reverse transcription was done by mixing the reaction gently and centrifuging briefly. After which the reaction was incubated at the following temperatures, 25°C for 2 min, then 55°C for 20 min and 95°C for 1 min, to inactivate the reverse transcriptase enzyme. The synthesized cDNA was then stored at −20°C until use in qPCR.

### 2.4 Quantification of MBL Serum Protein Concentration

Serum samples from selected South African chicken breeds were stored at −80°C. MBL concentration in serum samples was measured using the ELISA sandwich assay with antibody–MBL antigen chicken MBL‐C (ADL, America), which contains a 96‐well test plate consisting of standards of known MBL concentrations, wash buffers, an MBL antigen and a biotinylated monoclonal antibody specific for MBL, an enzyme (streptavidin‐peroxidase) and a substrate solution. Serum samples from South African chicken breeds and standards of known MBL concentrations were loaded into the wells on a test plate containing 50 µL of each. The MBL antigen and the biotinylated monoclonal antibody specific for MBL were added to each well and incubated at 37°C for 60 min. The wells were washed, after which the enzyme, streptavidin‐peroxidase, was added according to the manufacturers. After incubating at 37°C for 30 min, the wells were washed to remove unbound enzymes, after which the substrate solution, which was reacted with the bound enzyme to induce a colour, was added. The intensity of the colour was proportional to the concentration of MBL protein present in the serum samples. The intensity of the colour was measured with an ELISA reader at 450 nm and was subsequently converted to the MBL concentration.

### 2.5 Quantitative PCR

qPCR was subsequently performed in 96‐well plates with Luna Universal Probe qPCR Master Mix (New England Biolabs, Ipswich, MA, USA) using a probe‐based qPCR assay. Each reaction contained 1 µL of cDNA template forward primer (10 µM), 0.5 µL reverse primer (10 µM), 0.5 µL TaqMan probe (10 µM), 0.5 µL TaqMan Universal PCR Master Mix (10 µL) and nuclease‐free water (20 µL); the primers used for this study are in Table 1. All the protocols are according to the manufacturer's information. The cycling procedure started with initial denaturation at 95°C for 10 min, and then denaturation at 95°C for 15 s, followed by annealing or extension at 60°C for 60 s with 40 cycles. The reactions were run on a CFX96 Real‐Time PCR System (Bio‐Rad) following a standard two‐step PCR program as suggested by the Luna Universal Probe qPCR Master Mix manual. Three technical replicates were run for each cDNA sample. The qPCR instrument generated a Ct (threshold cycle) value for each sample. Amplification of different input templates was evaluated based on the quantification cycle (Cq) value by implementing the ΔΔCt method. The relative expression of genes of interest was calculated by the ‘delta Ct’ (ΔΔCt) method. The delta Ct (ΔΔCt) method was used without efficiency correction: ΔCt1Ct (target A treated) − Ct (ref B treated). ΔCt2Ct (target A control) − Ct (Ref B control). ΔΔCtΔCt1 (treated) − ΔCt2 (control). Final = 2^−(ΔΔCt)^. Then the fold change was derived from 2^−ΔΔCt^. The fold change was used to measure the changes in relative gene expression, indicating how many times the MBL gene expression has increased or decreased with respect to control:

$$\mathrm{Mathematically},\mathrm{Fold}\;\mathrm{change}={2^{-}}^{\Delta \Delta \mathrm{Ct}}$$

**TABLE 1:** Sequences of primers used for mannose‐binding lectin (MBL) mRNA gene analysis.

Target	Primer/Probe		Sequence	5′ Label	Length (bp)	PCR product size (bp)	References
MBL	F	Forward primer	TCCAGCCATCTTCTGTTTCAATTC	FAM	24	120	Kjærup et al. (2013)
MBL	R	Reverse primer	CCACAGATCAGCACCTTTACCT	FAM	22		
MBL	P	Probe	TGGCTGTCAGGAAAGCCAATG	FAM	21		
GAPDH	F	Forward primer	CCATGGAGAAGGTTGGGGTT	HEX	20	140	Zhang, Van Eijk, et al. (2017)
GAPDH	R	Reverse primer	CAAAGTTGTCATGGATGACC	HEX	20		
GAPDH	P	Probe	CCCACTCCTCCACCTTTGACGCTG	HEX	23		

The list of primers used in the study for assessing the mRNA expression of MBL is shown in Table 1.

### 2.6 Statistical Analysis

Data were analysed using the General Linear Model on MINITAB 17 software (Minitab, LLC 2017). Two‐way ANOVA was performed between MBL (independent variable) and breed (dependent factor); age was fitted as a covariate, and Tukey's multiple comparison post hoc test was used *(*p* ≤ 0.05) and ***(*p* ≤ 0.001).

The following statistical model was used:

$$Y_{ij}=\mu +B_{i}+\varphi j+\varepsilon_{ij}$$

where $Y_{ij}$ is the measurement of MBL response; *µ* is the MBL protein concentration; *B_i_* is the breed effect on MBL; 𝜑𝑗 is the covariate effect on age; and *Ɛ_ij_* is the random error variation.

## 3 Results

### 3.1 The Mean MBL Protein Concentration of Chicken Breeds in South Africa

**FIGURE 2:**
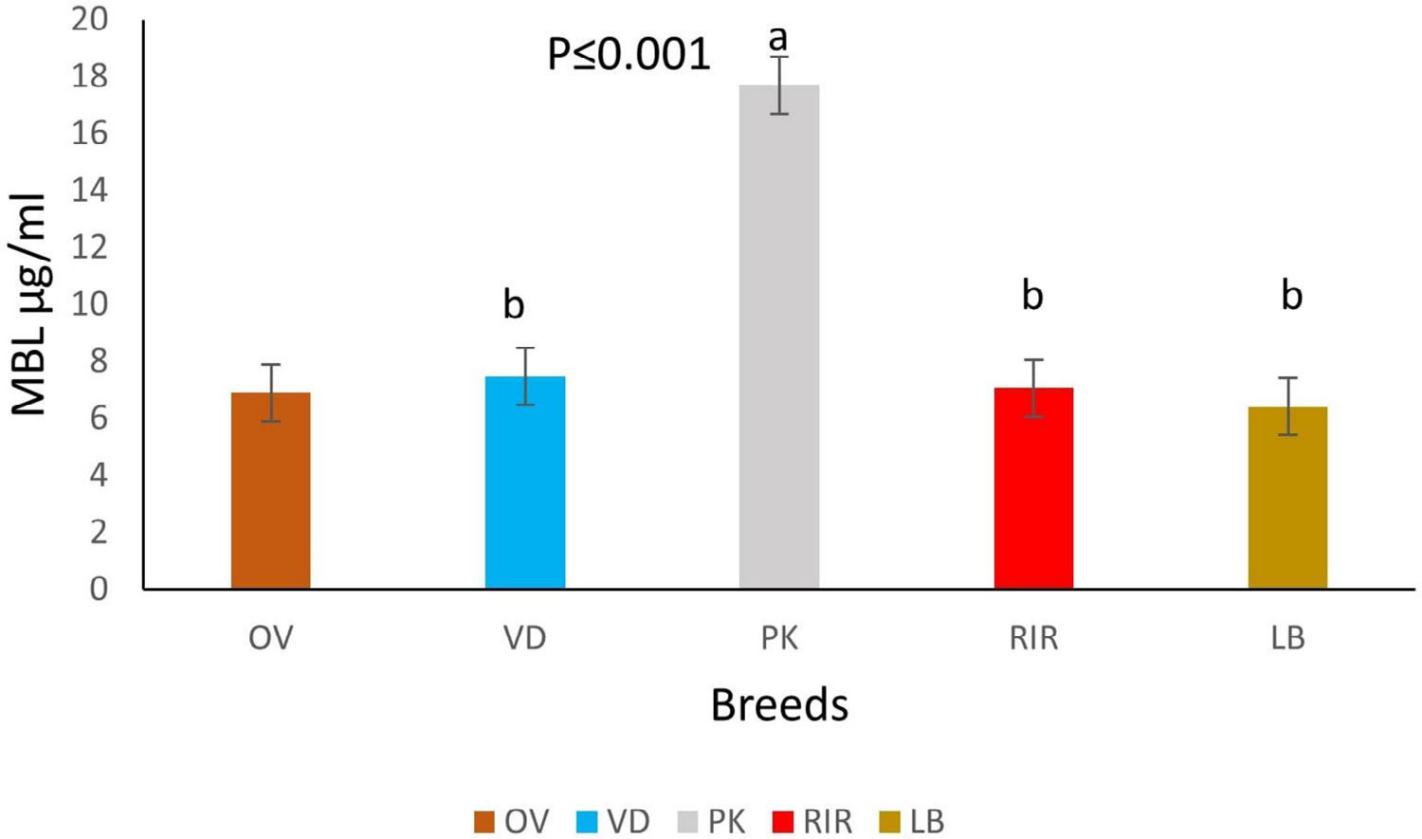
Mean protein concentration (with 95% CI) of mannose‐binding lectin (MBL) in the serum of five chicken breeds: Ovambo (OV), Venda (VD), Potchefstroom Koekoek (PK), Rhode Island Red (RIR) and Lohmann Brown (LB). Letters (a) and (b) indicate statistically significant differences ***p ≤ 0.001 between MBL protein concentration in chicken breeds in South Africa.

**FIGURE 3:**
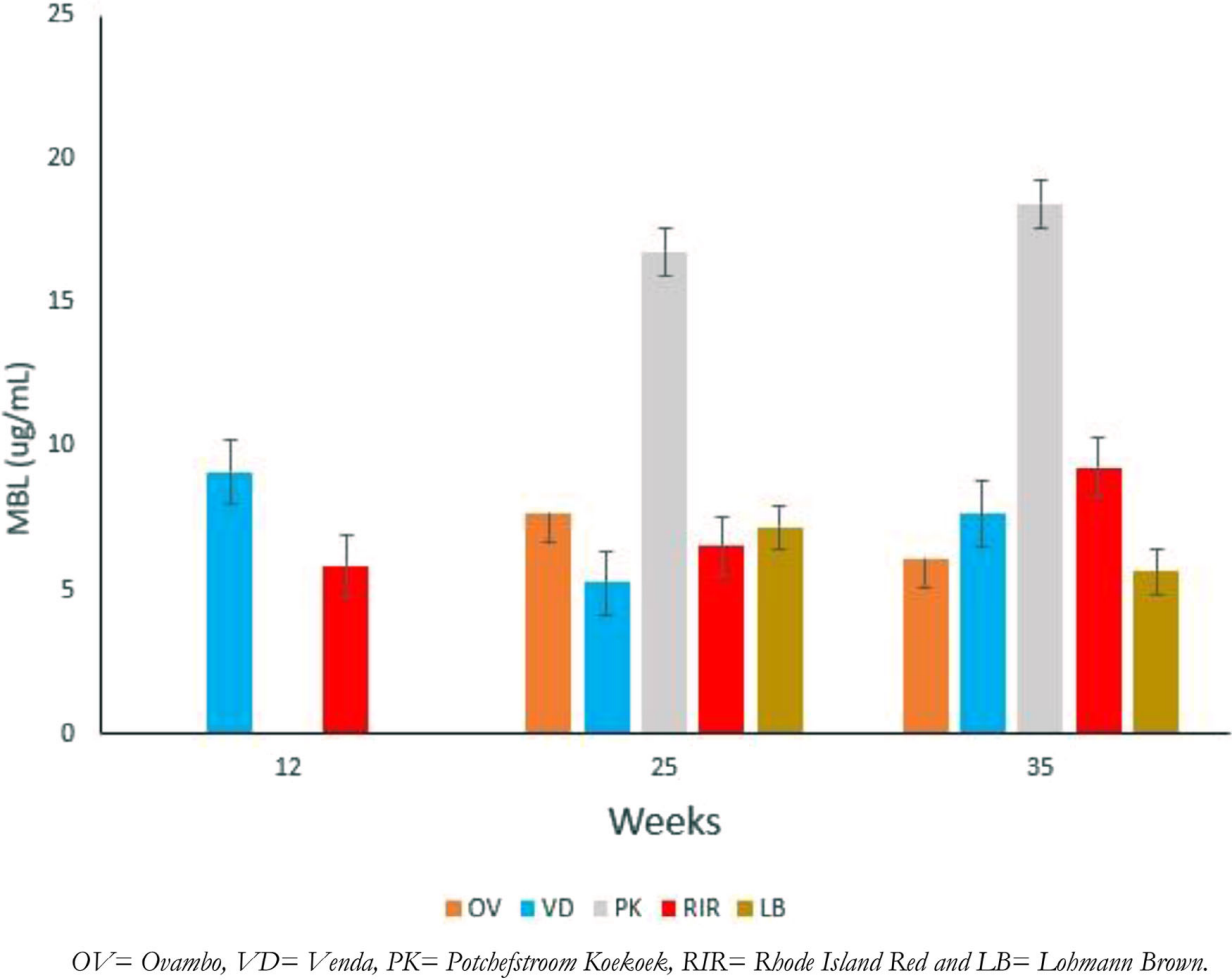
Mean concentration (with 95% CI) of mannose‐binding lectin (MBL) in the serum of five chicken breeds of OV, VD, PK, RIR and LB breeds, at week 12, 25 and 35 weeks. The mannose‐binding lectin expression of selected South African chicken breeds. The mean protein concentration of cMBL in selected South Africa chicken breeds at different age groups.

The findings from this study revealed a wide range of MBL protein concentrations in studied South African chickens, with a mean of 9.1 µg/mL and a range of 6.4–17.7 µg/mL (Table 2). Statistically significant differences (*p* < 0.001) were was observed between the indigenous chicken breeds. The serum MBL concentration of OV (6.89 ± 0.22 µg/mL) was lower than that of VD (7.46 ± 0.14 µg/mL) and PK (17.7 ± 0.21 µg/mL) (Figure 2). Among the exotic breeds, the genetic mean protein concentration of MBL in LB was 6.4 ±0.31 µg/mL, which was slightly lower than RIR 7.06 ± 0.3 µg/mL. No significant difference (*p* > 0.05) observed between the two exotic breeds (Figure 3).

**TABLE 2:** Mean and standard error mean of breeds and mannose‐binding lectin (MBL) protein concentration of selected South African chicken breeds.

				95% CI	
Breeds	*N*	Mean	SEM	Lower bound	Upper bound
OV	5	6.89^a^	0.22	6.878	8.482
VD	5	7.46^a^	0.14	4.478	6.082
PK	5	17.70^b^	0.21	15.990	17.594
RIR	5	7.06^a^	0.30	5.252	6.856
LB	5	6.40^a^	0.31	6.444	8.048

*Note*: Means with same letters are not significantly different from each other (*p* ≤ 0.005).

Abbreviations: LB = Lohmann Brown; OV = Ovambo; PK = Potchefstroom Koekoek; RIR = Rhode Island Red; SEM = standard error of mean; VD = Venda.

Figure 2 shows the protein concentration of MBL in the serum of five different chicken breeds (OV, VD, PK, RIR and LB) at different age groups. All the sampled chicken breeds were found to have statistically significant higher mean serum MBL concentration at Week 25 compared to Week 35, except the OV and LB breeds (*p* < 0.05). At Week 12, only VD (9.1 ± 0.15 µg/mL) and RIR (5.854 ± 0.44 µg/mL) serum concentrations were observed.

**FIGURE 4:**
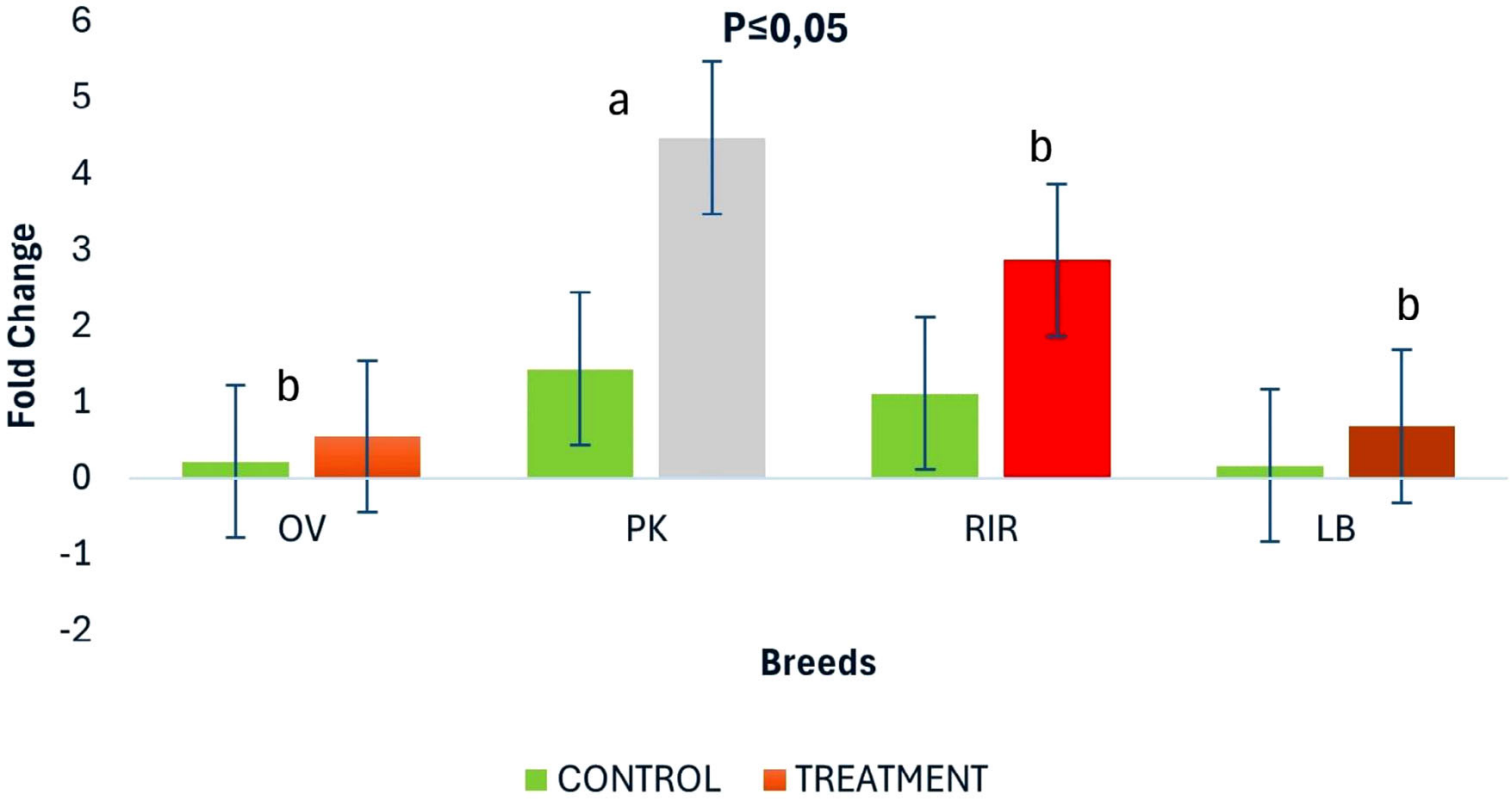
Differences in MBL expression (fold change) in chicken liver, with chicken with no presence of *Mycoplasma gallisepticum* serving as control. Letters (a) and (b) indicate statistically significant differences (*p* < 0.05) between MBL mRNA expression in chicken breeds. *p ≤ 0.05. LB = Lohmann Brown; MBL = mannose‐binding lectin; OV = Ovambo; PK = Potchefstroom Koekoek; RIR = Rhode Island Red.

The MBL expression was measured in the chicken livers and calculated as a fold change (40 − Ct values) compared to the endogenous control (Figure 4). The OV and LB breeds produced a 1‐fold decrease in MBL expression relative to that in the control group (*p* < 0.05), the RIR breed produced a 2‐fold increase in MBL expression relative to that in the control group (*p* < 0.05), and the PK breed produced a 3‐fold increase in MBL expression relative to that in the control group (*p* < 0.05).

The MBL expression in the liver was determined by qPCR in the OV, VD, PK, RIR and LB breeds (Figure 4). This was achieved using more complex relative quantification methods, which can be summarized as 2^−ΔΔCt^. Significant differences (*p* < 0.05) were detected between the control and the PK (*p* < 0.05) breed, whereas no significant difference (p > 0.05) was observed between the control and the other chicken breeds. The highest chicken MBL expression was observed in the PK breed among the other selected chicken breeds.

## 4 Discussion

The main purpose of this study was to detect, quantify and determine the expression of MBL protein and its expression in selected South African chicken breeds. Studies have shown that one of the important mechanisms of the innate host defence mechanism is the complement system (Idowu, Idowu, et&amp;#x000A0;al. 2021; Kjærup et al. 2013; Laursen, Nielsen, and Koch 1998; Ulrich‐Lynge et al. 2016; Zhang, Bouwman, et al. 2017). This mechanism ensures the homeostasis and activation of the innate immune system via the three pathways—classic, alternative and lectin pathways (Matsushita, Endo, and Fujita 2023). Moreover, the activities of MBL (binding to the mannose region of the pathogens to engulf and eliminate pathogens) are initiated by the lectin pathway (Idowu, Idowu, et al. 2021). The serum MBL concentration and its mRNA expression of studied chicken breeds in South Africa were successfully quantified.

This study revealed the presence and wide variation in MBL concentration among the studied chicken breeds in South Africa. The mean MBL level in chickens ranges from 6.4 ± 0.31 to 17.7 ± 0.21 µg/mL concentration. The observed differences in MBL protein concentration in the sampled breeds signifies that the chicken breeds with high MBL protein concentration activate the complement system faster than chicken with low MBL level (Zhang et al. 2017). To further explain the impact of MBL variation in chicken, differences in MBL level are a function of the genetic factors which vary among chicken breeds. Similar variations in MBL protein concentrations were observed by Norup et al. (2019), Kjærup et al. (2013), and Mamu et al. (2014). Furthermore, PK breeds had the highest MBL protein concentration in this study; this could be due to selective breeding that PK breeds had undergone for years. One of the criteria for the formation of PK breeds by breeders was careful selection of chickens with high immunity, which are less prone to diseases commonly associated with chickens (Habte et al. 2021).

To further explore the impact of age on MBL protein concentration, we quantified MBL protein at different age groups, namely 12, 25 and 35 weeks. The difference in the MBL protein concentration observed in this study at different age groups is analogous to previous findings of chickens at different age groups (Ulrich‐Lynge et al. 2015a). The disparity in MBL protein concentration across different ages of the same breed explained that MBL is upregulated at the early age of chickens, when the immune system is most vulnerable (Kjærup et al. 2013). At the time of exposure to different unknown diseases across the farm, MBL becomes upregulated upon exposure to diseases (Laursen and Nielsen 2000; Norup et al. 2019; Ulrich‐Lynge et al. 2015a). Song et al. (2021) observed that as age increases, immune cells become more developed, such that the maternal IgG levels deteriorate, after which chickens develop their own adaptive immune system. Moreover, it has been reported that MBL is among the most significant innate immune system mediators at the early developmental stage of the adaptive immune system in chickens (Laursen and Nielsen 2000; Lo and Woodruff 2020). Therefore, at different ages, the state of the maturity of the immune cell and the nature of MBL, among other factors, are responsible for the variation in MBL protein concentration in chickens.

To quantify MBL expression of chicken MBL, liver samples were collected from four different chicken breeds. Previous studies have shown that MBL mRNA can be expressed in different organs in chickens, such as the liver (Zhang, Bouwman, et al. 2017), oviduct (Jung et al. 2011), yolk sac (Cortés‐Coronado et al. 2017), ceca (Ulrich‐Lynge et al. 2015a) and lungs (Norup et al. 2019). However, for this study, MBL expression was quantified from liver samples only. This study observed the highest level of expression of MBL in PK breeds among all sampled breeds. This could be a response of MBL in combating MG in the sampled chicken breeds. On the basis of this finding, exposure to disease is one of the causes of variations in MBL protein concentration. This difference could be attributed to the ability of MBL to initiate the innate host defence mechanism at the entry of bacterial pathogens. Ulrich‐Lynge et al. (2015a) observed an upregulation of MBL at the entry of *S. enterica* in chicken breeds. This was attributed to the interaction of MBL with target organs acting as an acute phase reactant. MBL is known for its phagocytosis and opsonization characteristics.

It is important to note that chickens are more prone to disease at an early age due to a lack of colonization of the gut microbiota (Shehata et al. 2021). The possibility of a large population of bacterial species at the hatchery and pen usually affects the ability of the liver to secrete MBL. Arab, Martin‐Mateos, and Shah (2018) revealed that increase in the gut microbiota population can influence the development of liver disease due to the migration of microbes from the gut region to the liver. In this study, differences in MBL level were observed; this could be attributed to the health state of the liver organ in different sampled chicken breeds.

Furthermore, studies have shown that the presence of some bacteria like *Escherichia coli and Salmonella* spp. influences the upregulation of some immune‐related proteins such as MBL (Denzer, Schroten, and Schwerk 2020; Ulrich‐Lynge et al. 2015b; Zheng et al. 2021). The functional traits of the hepatic region (liver) in expressing innate defence proteins such as MBL at the time of exposure to disease cannot be overemphasized (Zheng et al. 2021). The liver expresses MBL directly to inactivate bacteria, hence preserving the chickens’ tissue against injury or damage. MBL itself does not recruit cells, but it aids in opsonization (the process by which pathogens are identified for phagocytosis), or the engulfing and digesting, by immune system cells such as neutrophils and macrophages. The complement system is a group of proteins that improves the immune response and is activated when MBL interacts with pathogens (Świerzko and Cedzyński 2020). It is important to note that additional immune cells such as macrophages are drawn to the infection site due to the activation of the complement system through lectin pathway to respond against invading pathogens (Coumou et al. 2019; Mu et al. 2020). These factors make them important components of the chicken's resistance against infection.

This study observed interbreed variation in MBL expression between the four breeds sampled. The PK chicken breed MBL expression was two to three times greater than that of the LB and OV breeds (Norup et al. 2019). The difference in the fold change observed in this study is comparable to previous findings in the chicken liver (Ulrich‐Lynge et al. 2015a). It can be said that PK breeds showed a greater humoral immune response to *M. gallisepticum* than in other chicken breeds. This could be due to heterosis (hybrid vigour) found in the PK breeds, which could influence the increase in chicken MBL in comparison to other chicken breeds (Maghsoudi et al. 2015). Additionally, higher MBL expression in PK breeds could be attributed to the presence of A3 alleles responsible for high MBL (Kjærup et al. 2014), whereas the lower expression of MBL in OV and LB breeds could be due to the presence of A1 alleles (Kjærup et al. 2014). Apart from age impact on MBL expression variation, stress (Schou et al. 2010) and the type of bacteria (gram‐negative or gram‐positive) (dos Santos Silva et al. 2019) could influence MBL expression. It is important to note that there are several pathways for MBL expression in chicken. Such pathways are Toll‐like receptors (TLR) pathways; in chickens, TLR2 and TLR4 are known to be associated with the regulation of MBL expression after the activation of the TLR signalling (Smith and Fiddaman 2022). Another pathway is cytokine‐mediated pathway; this pathway modulates the MBL expression in chicken upon the entry of bacteria with the stimulation of interleukin‐1 (IL‐1) and interferon‐gamma (IFN‐γ) (Oosting et al. 2016). Lastly, pathogen‐specific activation is another pathway that stimulates the expression of MBL; this pathway can be induced by bacteria lipopolysaccharides for bacteria (dos Santos Silva et al. 2019). Hence, any of these pathways with complement system activation which have been explained previously could be responsible for the expression of MBL in the chicken breeds sampled.

Several studies had validated the importance of MBL in disease resistance and susceptibility. First, the dysfunction of the activation of the complement system, such as the lectin pathway, of which MBL is a crucial member, led to an increase in faecal shedding containing bacteria (Ulrich‐Lynge et al. 2016). Moreover, Ulrich‐Lynge et al. (2016) observed a positive correlation between MBL expression and chicken susceptibility to infectious diseases. Similarly, it can also be said that the ability of the MBL to influence cytokine production, which in turn helps to control inflammations in chickens by allowing immune system to create an immune defence mechanism against bacteria (Schou et al. 2010). Lastly, studies have shown that the higher the MBL protein and expression in chickens, the higher the chances of chicken combating bacterial diseases, and this should be a great consideration in breeding plans with less or no use of antibiotics, which could lead to antimicrobial resistance (Schou et al. 2010; Ulrich‐Lynge et al. 2016).

## 5 Conclusion

This study observed the presence of varying quantities in cMBL protein concentration in the sampled South African chicken breeds. Moreover, it was discovered that at different weeks, the level of MBL protein varies across different breeds. This highlights the genetic diversity of MBL in sampled South African chicken breed populations. Furthermore, the remarkable difference in MBL expression in the presence of *M. gallisepticum* among the sampled South African chicken breeds was observed. Therefore, it can be concluded that high MBL concentration in chicken with other innate immune proteins could assist in preventing the incessant usage of antibiotics in chicken production and the spread of *M. gallisepticum*. Future studies should consider potential confounding factors that could have influenced the MBL protein and its expression such as stress and feed formulation in the South African chicken breeds.

## Author Contributions

**P.A.I**. and **B.M**.: conceptualization. **P.A.I**., **T.J.N**., and **B.M**.: methodology. **P.A.I**. and **T.J.N**.: software. **P.A.I**., **T.J.N**., and **B.M**.: validation. **P.A.I**., **T.J.N**., and **B.M**.: formal analysis. **P.A.I**. and **T.J.N**.: investigation. **P.A.I**., **T.J.N**., **K.A.N**., and **B.M**.: resources. **P.A.I**., **T.J.N**., and **B.M**.: data curation. **P.A.I**.: writingoriginal draft preparation. **P.A.I**., **O.T.Z**., **T.J.N**., **K.A.N**., and **B.M**.: writingreview and editing. **P.A.I**., **T.J.N**., and **B.M**.: visualization. **P.A.I**., **O.T.Z**., **T.J.N**., **K.A.N**., and **B.M**.: supervision. **P.A.I**., **T.J.N**., **K.A.N**., and **B.M**.: project administration. **T.J.N**., **K.A.N**., and **B.M**.: funding acquisition. All authors have read and agreed to the published version of the manuscript.

## Ethics Statement

The study was approved by the Animal Research Ethics Committee of the Faculty of Science, Tshwane University of Technology AREC2021/10/002. Also, the study is in accordance with Animal Research: Reporting of In Vivo Experiments (ARRIVE) guidelines (https://arriveguidelines.org). Ethical rules guiding the local animal welfare regulations, practices, and management in conformity to ethical guidelines for animal usage in research by the Tshwane University of Technology were strictly adhered to.

## Conflicts of Interest

The authors declare no conflicts of interest.

## Data Availability

The article contains the original data and contributions made in this study; for more information, contact the corresponding author or authors.
